# Sex-Related Differences in Life Expectancy Compared to General Population after Surgery for Ascending Aortic Aneurysm

**DOI:** 10.3390/jcm13154554

**Published:** 2024-08-04

**Authors:** Marcel Almendárez, Francesco Formica, Jorge Gutierrez Sáenz de Santamaría, Pablo Avanzas, Alain Escalera, Rut Alvarez-Velasco, Isaac Pascual, Jacobo Silva, Rocío Díaz, Alberto Alperi, Daniel Hernández-Vaquero

**Affiliations:** 1 Heart Area, Hospital Universitario Central de Asturias, Avenida de Roma S/N, 33011 Oviedo, Spain; marcel.almendarez@gmail.com (M.A.); alain_2623@hotmail.com (A.E.); rutalvarez3@gmail.com (R.A.-V.); ipascua@live.com (I.P.); jsilva8252@yahoo.es (J.S.); diazmendezro@gmail.com (R.D.); alberto.alperi.garcia@hotmail.com (A.A.); dhvaquero@gmail.com (D.H.-V.); 2 Research Institute of the Principado de Asturias, Avenida de Roma S/N, 33011 Oviedo, Spain; 3 Department of Medicine and Surgery, University of Parma, 43121 Parma, Italy; francesco.formica@unipr.it; 4 Department of Medicine, Faculty of Medicine, University of Oviedo, 33006 Oviedo, Spain; jorge.gssm@gmail.com; 5 CIBER Cardiovascular, Instituto de Salud Carlos III, 28029 Madrid, Spain

**Keywords:** ascending aortic aneurysm, relative survival, aortic aneurysm surgery

## Abstract

**Background/Objectives**: Understanding sex-based differences in cardiovascular outcomes is paramount to improving clinical outcomes. Surgery is an aggressive but effective therapy for ascending aortic aneurysm. We sought to determine if being a woman is a risk factor for long-term mortality after this surgery. We compared their life expectancy with a general population of the same age, sex, year, and region. **Methods**: We compared men and women undergoing AAA surgery at our institution from 2000 to 2019. After balancing the population with propensity score (PS) matching, we compared long-term mortality control with a Cox regression. We determined the RS using the Ederer II method and compared it to a healthy reference population of the same age, sex, and region. **Results**: From 2000 to 2019, 232 women and 506 men underwent ascending aortic aneurysm surgery. After a mean follow-up of 51.5 ± 34.5 months, sex was not an independent risk factor for long-term mortality in the multivariable analysis [HR: 0.68 (95% CI 0.43–1.07, *p* = 0.23)]. Matching by baseline characteristics, 196 pairs were analyzed with no differences regarding mortality in the Cox regression [HR: 1.11 (95% CI 0.65–1.9, *p* = 0.23)]. Men and women who survived the postoperative period presented a relative survival of 100.3% (95% CI 97.4–101%) and 100.3% (95% CI 98.9–101.1%), respectively, similar to the reference population without the disease. **Conclusions**: For patients undergoing AAA surgery, sex was not an independent predictor of mortality. Men and women who survived the postoperative period presented a similar life expectancy to that of the reference population (people free from the disease of the same age, sex, year, and region).

## 1. Introduction

Numerous sex-related differences in cardiovascular disease regarding prevalence, clinical outcomes, and treatment have recently been documented. There is a particular interest in coronary artery disease and valve replacement surgery, where being a woman has been associated with increased short- and long-term mortality. Such differences have incited a sex-based approach to cardiovascular research to improve clinical care [1,2,3,4].

Aortic aneurysms are the second most prevalent arterial disease after atherosclerosis, with a current prevalence of ascending aortic aneurysms (AAA) of 8 in 100,000 patients per year and an annual death rate of 7.7 per 100,000 patients in Europe [5,6]. Clinical representation of female patients is relatively scarce due to the low prevalence of this disease in women. The presentation of the disease may vary according to sex, with a later diagnosis in women that may lead to different outcomes. There is evidence suggesting that women may have a more aggressive form of the disease, with faster dilations of the aorta and a higher dissection rate. Furthermore, heritable AAA has been reported in up to 25% of Turner syndrome and x-linked disorders [7]. In acute aortic syndrome, female patients had a larger ascending aortic diameter than males, suggesting a different timing in acute events.

Only a handful of studies have analyzed sex-related outcomes after AAA surgery, with different results [8,9,10]. These studies contain contradictory information due to significant heterogeneity, including patients with acute aortic syndrome and elective surgeries, others performing only univariable analyses, relatively short follow-up periods, and small sample sizes. A recent analysis of aortic arch surgery has shown differences in mortality trends with a reduction through the study period for female patients with no changes observed in men [11]. Moreover, significant differences in the baseline characteristics of male and female patients warrant further sensitivity analysis, such as employing a propensity score to account for confounding and selection bias. A large analysis of 1148 patients showed similar mortality rates during the hospitalization period and in a median follow-up of 7.1 years after matching [12].

A study by our group reported that patients undergoing elective surgery for AAA who survived the postoperative period completely recovered their life expectancy in the long-term follow-up. However, there was no analysis according to sex [13]. Women live longer than men in the general population. Thus, it is especially relevant to know if women undergoing AAA surgery recover their life expectancy after correcting the defect.

Our objective was to determine if being a woman is a risk factor for long-term mortality after AAA surgery and to know if men and women recover their life expectancy after surgery, compared to the general population of the same age, sex, year, and region.

## 2. Methods

### 2.1. Study Design

This is a retrospective analysis of our institutional database of all cases of patients who underwent AAA surgery from 2000 until 2019. We included subjects who underwent isolated ascending aortic replacement and other concomitant aortic procedures. Ethical approval was obtained from the corresponding IRB (reference number: 20/087). This article’s data will be shared upon request.

Exclusion criteria were patients < 18 years old, acute aortic syndrome, previous aortic root or ascending aorta surgery, and concomitant mitral or tricuspid surgery.

Data recollection for the baseline characteristics, procedure, hospitalization, and discharge were obtained from a dedicated database prospectively collected. Follow-ups were performed by accessing and receiving data from the electronic clinical history and phone interviews for patients needing more information.

We constructed our reference population using mortality tables from our region provided by the National Institute of Statistics of Spain (INE) [14]. This institution publishes high-quality tables stratifying mortality by age, sex, year, and region in Spain. Standardized endpoints and causes of death were defined according to the Academic Research Consortium-2 consensus document [15].

We stratified all analyses according to sex. To compare relative survival to the general population, we repeated the estimations on patients who survived the postoperative period. We considered this period 30 days after the intervention or until hospital discharge if it surpassed 30 days.

### 2.2. Endpoints

The primary endpoint of this study was to determine if being a woman is a risk factor for long-term mortality among patients who underwent elective AAA surgery. The secondary endpoint was to compare the life expectancy of men and women who underwent elective AAA surgery and survived the postoperative period to that of the general population of the same age, sex, and region.

### 2.3. Statistical Analyses

Categorical variables were described as n (%) and quantitively as mean ± SD. Categorical variables were compared with Fisher’s exact test and quantitative variables with Student’s T-test. We calculated standardized differences since *p*-values may vary according to sample size [16]. Data distribution was evaluated using the Shapiro–Wilk test.

Stratifying by sex, we estimated our sample’s observed survival (OS), which was calculated with the usual Kaplan–Meier method, and we compared it to the expected survival (ES) of the reference population estimated with the Ederer II method. ES is the survival probability of a population like the studied sample (with the same age, sex, and region) but free from the underlying disease (i.e., AAA). We obtained the data from the National Institute of Statistics, providing mortality tables from subjects of the same age, sex, year, and geographical region. If the ES is included in the OS’s 95% confidence interval (CI), no statistical differences are considered to exist [17,18]. We calculated the relative survival (RS) from these estimations, defined as the ratio between the OS in our sample during a specific time interval and the ES of the reference group. In other words, it refers to the likelihood of patients surviving if they were to succumb to the disease (AAA), the subsequent surgery, or its consequences. If the 95% CI of the RS includes 100%, there would be no evidence of mortality due to the AAA or the surgery. In other words, there is no excess of mortality. Therefore, the treatment provided effectively leads to a life expectancy similar to that of the general population [19].

We performed a multivariable Cox regression to determine the factors that predicted mortality. The variables introduced were defined using a backward stepwise with a cutoff *p*-value < 0.10. Associations were expressed as hazard ratios (HRs) with a 95% confidence interval (95% CI). The model’s predictive capacity was evaluated with Harrel’s C test, and survival was evaluated using the Kaplan–Meier curves. The differences were statistically significant, with a *p*-value of <0.05.

Finally, to determine the influence of female patients on long-term mortality after AAA surgery, we performed a propensity score (PS) matched analysis. We included all baseline characteristics and calculated the propensity score with a multivariable logistic regression. We matched each woman to a man using the nearest neighbor method, specifically a 1:1 matching scheme, with no replacement, a caliper of 0.05, and a greedy matching approach. We compared baseline characteristics with standardized differences. An absolute value of >0.10 was considered significant. We used visual graphics to assess the diagnostics of the model. If a satisfactory balance among groups was obtained, survival curves using the Kaplan–Meier method were performed and compared with the stratified log-rank test. Hazard ratios were estimated with a Cox regression in the matched sample using men as the reference population [16,20,21]. Statistical analysis was performed with STATA 15 IC (StataCorp, College Station, TX, USA).

## 3. Results

From 2000 to 2019, 738 patients underwent AAA surgery and were included for analysis. From this sample, 232 patients were female (31.4%), and 506 were male (68.6%) (Supplemental Figure S1). The mean age was 65 years, which was similar across groups. Men had more cases of dyslipidemia [179 (35.4%) vs. 62 (26.7%)], stroke/transient ischemic attack (TIA) [26 (5.1%) vs. 2 (0.9%)], chronic obstructive pulmonary disease (COPD) [96 (19%) vs. 12 (5.2%)], and fewer cases of chronic kidney disease [112 (22.1%) vs. 80 (34.5%)]. The rest of the baseline characteristics can be consulted in Table 1.

In the procedural characteristics, the most frequently performed surgery was AAA replacement with concomitant aortic valve replacement (AVR) in more than half of the patients, using biological prosthetic valves in over 75% of the cases without sex differences. There were 40 women (17.2%) requiring circulatory arrest, compared to 44 men (8.7%). Cross-clamping time and cardiopulmonary bypass time were longer for males: 115.5 ± 53.6 vs. 105.1 ± 48.6 and 142.4 ± 62.4 vs. 132.2 ± 56, respectively. Details of procedural characteristics are shown in Table 1.

Reinterventions in the early postoperative period were needed in 46 men (9.1%) and 14 women (6%). In 16 cases, the main reason was severe pericardial effusion as a result of bleeding. A total of 30 men (68.2%) and 14 women (31.8%) died in the perioperative period, including the first 30 days after the surgery. Cardiogenic shock was the most frequent cause of death, followed by infection or sepsis. After the acute phase, reinterventions during the follow-up period were needed in 34 men (5.9%) and 24 women (10.3%). Causes for reintervention can be consulted in Supplementary Table S1.

**Figure 1:**
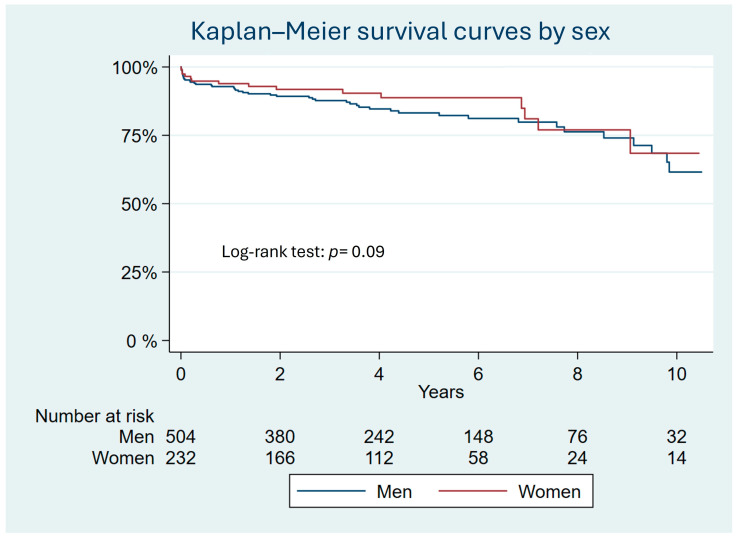
Kaplan–Meier curves of men and women who underwent ascending aortic aneurysm surgery showing similar mortality rates at the end of the follow-up period in the unmatched sample.

After the perioperative period, 66 males (13%) and 20 females (8.6%) died during the follow-up period. The most common causes of death were cancer and heart failure. There was a similar distribution according to sex. Detailed results of causes of death by sex can be consulted in Supplementary Table S2. Complications in the follow-up can be consulted in Supplementary Table S3. Multivariable Cox regression analysis shows that sex was not a predictor of mortality in the long-term follow-up (Table 2 and Figure 1).

### 3.1. Matched Sample

**Figure 2:**
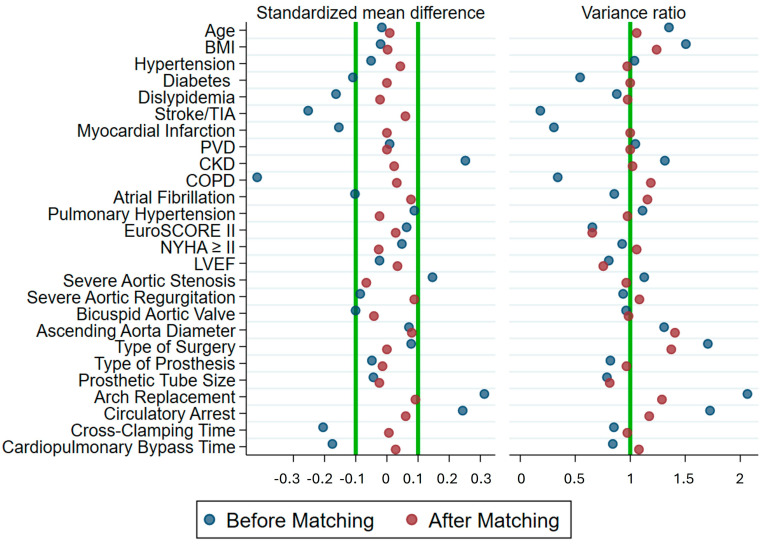
Balancing diagnostics of the baseline characteristics of men and women undergoing ascending aortic aneurysm surgery showing variables before (blue circles) and after (red circles) propensity score matching. The mean standardized difference after matching includes all the variables within the range of −0.1 to 0.1 (Green lines). Likewise, in the variance ratio, variables tend to be 1, indicating that the propensity score was effective in controlling differences among men and women.

The standardized difference showed imbalances in baseline characteristics, including diabetes, dyslipidemia, stroke/TIA, myocardial infarction, CKD, COPD, atrial fibrillation (AF), severe aortic stenosis, bicuspid valve, circulatory arrest, cross-clamping, and cardiopulmonary clamping time. After PS matching, 196 pairs were obtained, and standardized differences were analyzed; all variables were well-balanced (Table 3). The baseline characteristics used to perform the PS matching and balancing diagnostics are shown in Figure 2 and Supplementary Figures S2 and S3.

Complications during the hospitalization of the 196 pairs were analyzed, showing a tendency toward higher rates of permanent pacemaker implantation for women [14 (7.2%); *p* = 0.07] than men [6 (3.1%)]. There were no significant differences in deaths during the first 30 days (Table 4).

The mean follow-up was 51.5 months ± 34.5. Almost all patients presented a ≤ II NYHA functional class and a preserved left ventricle ejection fraction during the follow-up. Male patients had more cardiovascular readmissions (Table 4).

**Figure 3:**
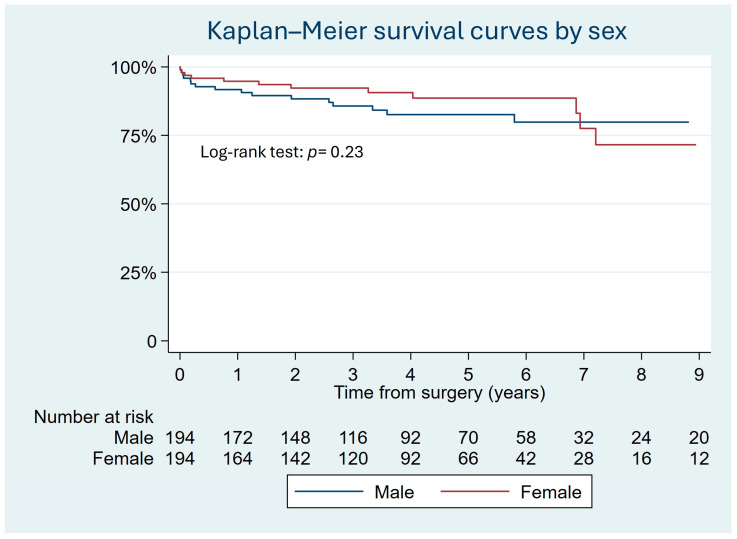
Kaplan–Meier survival curves show similar mortality for men and women at the end of the follow-up after ascending aortic aneurysm surgery in the matched sample.

Thirty men (15.3%) died in the matched sample compared to twenty-eight (14.3%) women. The Kaplan–Meier survival curves show that sex was not an independent risk factor for long-term mortality, as determined by the log-rank test (*p* = 0.23) (Figure 3). Cox regression analysis showed an HR of 1.11 (95% CI 0.65–1.9).

### 3.2. Relative Survival

**Figure 4:**
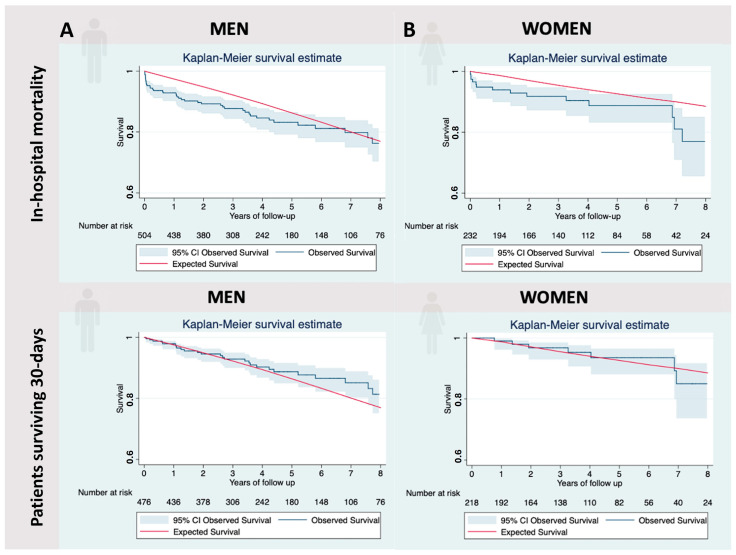
(**A**). Observed survival (OS) and expected survival (ES) for all men. The reference population’s ES (red line) is greater than our sample’s OS. After including men surviving the postoperative period, the ES is included within the 95% CI (blue sector) of the OS. (**B**). OS and ES for all women. The reference population’s ES (red line) is greater than our sample’s OS. After including women surviving the postoperative period, the ES is included within the 95% CI (blue sector) of the OS.

The mean follow-up for the censored observations was 52.7 ± 38.1 months, totaling 130 deaths (17.2%). For men, the OS was 92.3% (95% CI 90–94.3%), 80.6% (95% CI 76.1–86%), and 76.1% (95% CI 70.3–80.9%) for the first, sixth, and eighth years of follow-up, respectively. The ES was 94.7%, 83.2%, and 76.9% for the same period. After the sixth year, the ES was included in the 95% CI of the OS. However, for patients who survived the first 30 days, the OS and ES curves converged from the first year of follow-up: 97.8% (95% CI 96–98.8%) and 97.5%, respectively. The RS of the global sample who survived the first 30 days was 100.3% (95% CI 98.9–101.1%), indicating no excess mortality compared to the general population matched by sex, age, year of the event, and region (Table 5, Figure 4A).

Similar results were obtained for women OS and ES curves converging after the sixth year: 88.3% (95% CI 82.5–92.3%) and 91.2%, respectively. However, when considering only women who survived the first 30 days, the RS retrieved from the first year was found to be 100.3% (95% CI 97.4–101%) (Table 5, Figure 4B).

## 4. Discussion

The main findings of our study are that (i) sex was not an independent predictor of long-term mortality after AAA surgery, and (ii) men and women who survived the first 30 days after surgery presented a life expectancy without significant differences to the general population of their same age, sex, and region. Surgery seems utterly effective in restoring life expectancy for this subgroup of patients.

In contrast to recent studies, women had a similar age to men, yet men presented a higher rate of comorbidities such as dyslipidemia, stroke, and COPD [12]. The absolute aortic diameter was statistically significantly higher in women than men; however, when indexed to body surface area, it was significantly higher for women in consonance with modern studies [7].

### 4.1. Relative Survival after Ascending Aortic Aneurysm Surgery

RS is traditionally used to compare survival in cancer therapies [17,18]. Nevertheless, it has been previously described in the cardiovascular field [1,22,23]. However, this is the first time stratifying the RS of AAA surgery based on the long-term follow-up data according to sex. Mortality regarding AAA surgery varies according to different studies, some suggesting that women may have worse outcomes during the long-term follow-up, and others, when adjusting their model to baseline characteristics, have failed to demonstrate this result [8,10]. For this reason, we sought to determine if RS varied according to sex and if men recovered life expectancy earlier than women. Moreover, mortality in the general population varies significantly, with women living between 4 and 9 years longer than men [24].

Given the observational nature of our study, the Ederer II is the method of choice when estimating relative survival. Most observational studies analyzing mortality are unreliable in determining the cause of death of patients. However, this method compares our sample of patients with subjects from the general population who are of the same age, sex, year, and region and who are free from the disease. Our study does not need to know the causes of death because, from a theoretical point of view, the relative survival estimates the survival if patients could only die from the disease, the surgery, or its complications [17,18,19]. This is because a relative survival of 100% means no deaths are derived from the surgery or its complications.

The global sample of men and women shows a similar pattern where in-hospital mortality penalizes the RS during the first year. However, after this period, the RS of men and women stabilizes and remains equal to the reference population (Table 4). These findings expand upon the results published by Vaquero et al., confirming that if patients survive the first 30 days, they will fully recover their life expectancy, regardless of sex [13]. These results support intervening AAA irrespective of sex and encourage us to improve the postoperative period since mortality is directly influenced by complications during hospitalization, and surviving this period will confer an excellent long-term prognosis.

### 4.2. Sex as a Risk Factor for Long-Term Mortality

We found that sex was not an independent predictor of long-term mortality. Our results vary from those found by Beller et al., where female patients had an increased long-term mortality. However, they only performed a univariate analysis, not considering possible confounders [8].

The results of Voigt et al. indicate that there was initially a higher crude mortality rate among women. However, their results concur with ours when adjusting for potential confounders in a multivariable regression analysis [10]. Our sample was highly unbalanced, with significant differences in the baseline characteristics, showing the importance of controlling confounders. For this reason, we performed PS matching to obtain a balanced sample. After PS matching, including all baseline and procedural variables, we received a reasonable number of pairs and showed that sex was not an independent risk factor for long-term mortality. A recent analysis by Al-Tawil et al. of 1148 patients with a median follow-up of 7.1 years shows similar results to our study, with no differences regarding mortality in the long-term follow-up [12].

### 4.3. Limitations

This is an extensive database of more than ten years of follow-up of a single center, which is the institution’s reference for AAA surgery in our region. This is a limitation and a strength since our sample is more homogenous. Moreover, we did not include patients with acute aortic syndrome since the prognosis differs radically from elective surgeries. Furthermore, this work presents inherent biases of observational and retrospective studies with potential influence on the late outcomes of patients. Causes of death may not have been evident in all cases. However, we used a method that allowed us to overcome this possible bias by comparing and matching to the general population. Another significant limitation is the long duration of the study, which may lead to differences regarding surgical techniques, perioperative management, and the definitions utilized to indicate surgery and define perioperative complications.

## 5. Conclusions

In conclusion, after accounting for confounding factors through Cox regression and PS matching, it was determined that sex does not pose an independent risk for long-term mortality in patients who undergo elective AAA surgery.

For all men and women, the life expectancy of patients who undergo elective AAA surgery and survive the postoperative period is similar to that of the general population of the same age, sex, and region.

## Figures and Tables

**Table 1:** Baseline and procedural characteristics for men and women undergoing ascending aortic aneurysm surgery.

Variable	Male (n = 506)	Female (n = 232)	*p*	SD
Baseline Characteristics				
Age (years)	65.4 ± 12.4	65 ± 14.5	0.710	−0.02
Body mass index (kg/m^2^)	28.2 ± 4.2	28.1 ± 5.2	0.814	−0.02
Hypertension	342 (67.6)	150 (64.7)	0.433	−0.05
Diabetes mellitus	57 (11.3)	20 (8.6)	0.274	−0.11
Dyslipidemia	179 (35.4)	62 (26.7)	0.021	−0.16
TIA/Stroke	26 (5.1)	2 (0.9)	0.005	−0.25
Myocardial infarction	14 (2.8)	2 (0.9)	0.102	−0.15
Peripheral vascular disease	16 (3.2)	10 (4.3)	0.438	0.08
Chronic kidney disease	112 (22.1)	80 (34.5)	<0.001	0.25
COPD	96 (19)	12 (5.2)	<0.001	−0.41
Atrial fibrillation	107 (21.1)	42 (18.1)	0.348	−0.12
Pulmonary hypertension	116 (22.9)	59 (25.4)	0.516	0.09
EuroScore II	3.6 ± 3. 9	3.8 ± 3.1	0.491	0.06
NYHA functional class ≥ II	408 (80.6)	194 (83.6)	0.331	0.04
LVEF (%)	58.2 ± 38.8	55.5 ± 9.6	0.308	−0.02
Severe aortic stenosis	153 (30.2)	80 (34.5)	0.212	0.14
Severe aortic regurgitation	164 (32.4)	68 (29.3)	0.4	−0.08
Bicuspid aortic valve	212 (41.9)	84 (36.2)	0.14	−0.10
Ascending aorta diameter (mm)	50.7 ± 8.1	51.4 ± 9.1	0.278	0.07
Body surface area (m^2^)	1.9 ± 0.2	1.7 ± 0.2	0.005	−1.34
Ascending aorta diameter/BSA (mm/m^2^)	26.6 ± 0.2	30.6 ± 0.4	>0.001	0.66
Procedural Characteristics				
Type of surgery			0.310	0.07
Isolated AA replacement	52 (10.3)	34 (14.7)		
AA replacement and AVR	262 (51.8)	124 (53.5)		
Bentall–De Bono procedures	47 (9.3)	10 (4.3)		
AA replacement and aortic RR	110 (21.7)	30 (12.9)		
AA replacement and valve repair	15 (3)	8 (3.4)		
AA replacement and arch replacement	16 (3.1)	14 (6)		
AA and arch replacement and AVR	4 (0.8)	12 (5.2)		
Mechanical aortic prosthesis	118 (23.3)	54 (23.3)	0.9895	−0.04
Prosthetic tube size			0.404	−0.04
26	38 (7.5)	37 (15.9)		
28	123 (24.3)	86 (37.1)		
30	246 (48.6)	94 (40.5)		
32	79 (15.6)	12 (5.2)		
34	18(3.6)	3 (1.3)		
36	2 (0.4)	0		
Circulatory arrest	44 (8.7)	40 (17.2)	<0.001	0.24
Cross-clamping time (min)	115.5 ± 53.6	105.1 ± 48.6	0.011	−0.20
Cardiopulmonary bypass time (min)	142.4 ± 62.4	132.2 ± 56	0.034	−0.18

Variables are represented as mean ± standard deviation for quantitative variables and number (%) for categorical variables. Abbreviations—AA: ascending aorta; AVR: aortic valve replacement; BSA: Body surface area; LVEF: left ventricle ejection fraction; NYHA: New York Heart Association; RR: root remodeling; SD: standardized deviation; TIA: transient ischemic attack.

**Table 2:** Univariate and multivariable analysis of men and women undergoing ascending aortic aneurysm surgery.

Univariate Analysis				Multivariable Analysis			
Variable	HR	95% CI	*p*	Variable	HR	95% CI	*p*
Sex	0.78	0.52–1.17	0.230	Sex	0.68	0.43–1.07	0.092
Age	1.04	1.02–1.05	<0.001	Age	0.99	0.98–1.02	0.981
Hypertension	1.1	0.76–1.59	0.606	Stroke/TIA	1.64	0.75–3.58	0.213
Diabetes	1.28	0.77–2.12	0.327	CKD	2.46	1.56–3.89	<0.001
Dyslipidemia	0.89	0.62–1.31	0.567	Atrial fibrillation	2.04	1.32–3.18	0.001
Stroke/TIA	2.44	1.28–4.68	0.007	Pulmonary hypertension	1.05	0.63–1.76	0.842
Myocardial infarction	1.41	0.52–3.82	0.498	EuroScore II	1.07	1.03–1.11	<0.001
PVD	0.31	0.07–1.2	0.105	Ascending aorta diameter	1.03	1.01–1.06	0.005
CKD	2.76	1.95–3.91	<0.001	Arch replacement	2.08	0.37–11.5	0.404
COPD	1.62	1.06–2.48	0.025	Circulatory arrest	0.93	0.17–5.02	0.938
Atrial fibrillation	2.21	1.54–3.19	<0.001				
Pulmonary hypertension	1.47	0.99–2.2	0.06				
EuroScore II	1.08	1.05–1.11	<0.001				
NYHA ≥ 2	1.35	0.82–2.24	0.236				
LVEF	1.01	0.99–1.01	0.165				
Severe aortic stenosis	1.17	0.81–1.69	0.391				
Severe aortic regurgitation	1.02	0.7–1.48	0.915				
Bicuspid valve	0.75	0.52–1.08	0.120				
Ascending aorta diameter	1.05	1.03–1.07	<0.001				
Arch replacement	1.91	1.12–3.25	0.017				
Circulatory arrest	1.62	0.95–2.74	0.075				
Cross-clamping time	1.01	0.99–1.01	0.108				
Cardiopulmonary bypass time	1.01	1.00–1.06	0.108				

Abbreviations—COPD: chronic obstructive pulmonary disease; CKD: chronic kidney disease; LVEF: left ventricle ejection fraction; NYHA: New York Heart Association; PVD: peripheral vascular disease; TIA: transient ischemic attack.

**Table 3:** Baseline and procedural characteristics for men and women undergoing ascending aortic aneurysm surgery in the matched sample.

Variable Matched	Male (n = 196)	Female (n = 196)	*p*	SD
Baseline Characteristics				
Age (years)	64.2 ± 14.2	64.4 ± 14.7	0.933	0.01
Body mass index (kg/m^2^)	28.1 ± 4.5	28.1 ± 5	0.982	0.01
Hypertension	126 (64.3)	130 (66.3)	0.671	0.04
Diabetes mellitus	16 (8.2)	16 (8.2)	1	0
Dyslipidemia	54 (27.6)	52 (26.5)	0.820	−0.02
TIA/Stroke	0	2 (1.02)	0.156	0.06
Myocardial infarction	2 (1)	2 (1)	1	0
Peripheral vascular disease	6 (3.1)	6 (3.1)	1	0
Chronic kidney disease	56 (28.6)	58(29.6)	0.824	0.02
COPD	10 (5.1)	12 (6.12)	0.667	0.03
Atrial fibrillation	30 (15.3)	36 (18.4)	0.418	0.08
Pulmonary hypertension	52 (26.5)	50 (25.5)	0.818	−0.02
EuroScore II	3.7 ± 3.8	3.8 ± 3.1	0.781	0.02
NYHA functional class ≥ II	168 (85.7)	166 (84.7)	0.776	−0.02
LVEF (%)	55.5 ± 10.6	55.9 ± 9.2	0.736	0.03
Severe aortic stenosis	74 (37.8)	68 (34.7)	0.528	−0.07
Severe aortic regurgitation	54 (27.6)	62 (31.6)	0.376	0.09
Bicuspid aortic valve	82 (41.8)	78 (39.8)	0.681	−0.04
Ascending aorta diameter (mm)	50.2 ± 7.8	50.9 ± 9.2	0.421	0.07
Procedural characteristics				
Type of surgery			0.778	0
Isolated AA replacement	18 (9.2)	24 (12.2)		
AA replacement and AVR	108 (55.1)	110 (56.1)		
Bentall– De Bono procedures	12 (6.1)	10 (5.1)		
AA replacement and Aortic RR	42 (21.4)	30 (15.3)		
AA replacement and valve repair	8 (4.1)	6 (3.1)		
AA replacement and arch replacement	6 (3.1)	8 (4.1)		
AA and arch replacement and AVR	2 (1)	8 (4.1)		
Mechanical aortic prosthesis	60 (30.6)	50 (25.1)	0.802	−0.01
Prosthetic tube size			0.974	−0.02
26	12 (6.1)	28 (14.3)		
28	56 (25.9)	78 (39.8)		
30	96 (49)	76 (38.8)		
32	24 (12.2)	8 (5.1)		
34	8 (4.1)	4 (2)		
Circulatory arrest	20 (10.2)	22 (12.2)	0.522	0.06
Cross-clamping time (min)	108.8 ± 49.9	109.2 ± 49.2	0.984	0.01
Cardiopulmonary bypass time (min)	133.4 ± 55.8	135.1 ± 58	0.771	0.3

Variables are represented as mean ± standard deviation for quantitative variables and number (%) for categorical variables. Abbreviations—AA: ascending aorta; AVR: aortic valve replacement; LVEF: left ventricle ejection fraction; NYHA: New York Heart Association; RR: root remodeling; SD: standardized deviation; TIA: transient ischemic attack.

**Table 4:** Complications during the hospitalization and follow-up in the matched sample of men and women after ascending aortic aneurysm surgery.

Matched Variable	Male (n = 196)	Female (n = 196)	*p*
Hospitalization and 30-day outcomes			
Permanent Pacemaker	6 (3.1)	14 (7.2)	0.07
New Onset Atrial Fibrillation	28 (14.4)	34 (17.5)	0.41
Reintervention	26 (13.2)	16 (8.2)	0.1
Deaths	8 (4.1)	10 (5.1)	0.63
Follow-up			
NYHA ≤ II	164 (97.6)	166 (98.8)	0.78
LVEF	63.7 ± 10.2	59.2 ± 9.9	0.37
Readmission	12 (6.4)	4 (2.1)	0.04
Reintervention	20 (10.5)	12 (6.3)	0.14
Deaths	30 (15.3)	28 (14.3)	0.78

Variables are represented as mean ± standard deviation for quantitative variables and number (%) for categorical variables. Abbreviations—LVEF: left ventricle ejection fraction; NYHA: New York Heart Association.

**Table 5:** Relative survival of men and women of the global sample who survived the first 30 days.

Observed Survival, Expected Survival, and Relative Survival of All Men			
	Observed Survival	Expected Survival	Relative Survival
First year	92.3% (95% CI 90–94.3%)	97.4%	94.7% (95% CI 92–96.8%)
Second year	89.2% (95% CI 86.1–91.7%)	94.9%	99.3% (95% CI 97–100.7%)
Third year	87.2% (95% CI 83.8–89.9%)	92.2%	100.6% (95% CI 98.3–101.8%)
Fourth year	84.1% (95% CI 80.3–88.7%)	90.8%	99.5% (95% CI 96.5–101.2%)
Fifth year	82.6% (95% CI 78.5–86%)	86.3%	101.6% (95% CI 98.5–102.8%)
Sixth year	80.6% (95% CI 76.1–84.4%)	83.2%	101.2% (95% CI 97.3–102.8%)
Seventh year	79.4% (95% CI 74.6–83.4%)	80%	102.4% (95% CI 97.8–103.6%)
Eighth year	76.1% (95% CI 70.3–80.9%)	76.9%	99.7% (95% CI 92.9–102.4%)
Men Who Survived the first 30 days.			
First year	97.8 (95% CI 96–98.8%)	97.5%	100.3% (95% CI 98.5–101.3%)
Second year	94.5% (95% CI 91.9–96.3%)	94.9%	99.3% (95% CI 96.9–10.7%)
Third year	92.9% (95% CI 90–95%)	92.2%	101.1% (95% CI 99–102.1%)
Fourth year	90.2% (95% CI 86.7–92.8%)	89.4%	100.2% (95% CI 97.3–101.7%)
Fifth year	88.5% (95% CI 84.6–91.5%)	86.4%	101.6% (95% CI 98.4–102.8%)
Sixth year	86.4% (95% CI 81.9–89.8%)	83.3%	101.2% (95% CI 97.2–102.8%)
Seventh year	85% (95% CI 80.2–88.8%)	80.1%	102.4% (95% CI 97.7–103.6%)
Eighth year	81.4% (95% CI 75.3–86.1%)	77%	99.6% (95% CI 92.6–102.3%)
Observed survival, expected survival, and relative survival of all women			
	Observed survival	Expected Survival	Relative Survival
First year	93.7% (95% CI 89.6–96.2)	98.7%	95% (95% CI 90.8–97.5%)
Second year	92.7% (95% CI 88.4–95.5%)	97%	100.6% (95% CI 97.4–101.4%)
Third year	91.5% (95% CI 86.9–94.6%)	95.4%	100.3% (95% CI 96.6–101.3%)
Fourth year	90.1 (95% CI 85–93.6%)	94%	100% (95% CI 95.4–101.1%)
Fifth year	88.3% (95% CI 82.5–92.3%)	92.6%	99.5% (95% CI 93.7–100.1%)
Sixth year	88.3% (95% CI 82.5–92.3%)	91.2%	101.6% (95% CI 101.6–101.6%)
Seventh year	85% (95% CI 77.2–90.3%)	90%	97.5% (95% CI 86.9–100.4%)
Eighth year	75.8% (95% CI 63.7–84.4%)	88.5%	90.66% (95% CI 74.9–97.4%)
Women Who Survived the first 30 days.			
First year	99% (95% CI 96.1–99.8)	98.8%	100.3% (95% CI 97.4–101%)
Second year	96.8% (95% CI 93.1–98.6%)	97.1%	99.5% (95% CI 95.8–100.9%)
Third year	96.8% (95% CI 93.1–95.6%)	95.5%	101.7% (95% CI 101.7–101.7%)
Fourth year	95.3% (95% CI 90.7–97.6%)	94%	99.9% (95% CI 95.2–101.1%)
Fifth year	93.3% (95% CI 87.6–96.4%)	92.7%	99.4% (95% CI 93.4–101%)
Sixth year	93.3% (95% CI 87.6–96.4%)	91.2%	101.6% (95% CI 101.6–101.6%)
Seventh year	85.9% (95% CI 75.5–92.1%)	90%	93.3% (95% CI 81.2–98.2%)
Eighth year	85.9% (95% CI 75.5–92.1%)	88.6%	101.6% (95% CI 101.5–101.7%)

## Data Availability

This article’s data will be shared upon request.

## Supplementary Materials

The following supporting information can be downloaded at https://www.mdpi.com/article/10.3390/jcm13154554/s1. Figure S1: Flow chart of the study; Figure S2: Average treatment effect before and after matching showing similar probabilities among men and women; Figure S3: Box plot of the average treatment effect showing that the propensity score correctly balanced the baseline characteristics after matching; Table S1: Causes of reintervention; Table S2: Causes of death; Table S3: Complications during hospitalization and follow-up.
