# Supplementary material for: Sex-Related Differences in Life Expectancy Compared to General Population after Surgery for Ascending Aortic Aneurysm
Source: J Clin Med. 2024 Aug 4;13(15):4554. doi: 10.3390/jcm13154554 (PMC11313614; doi:10.3390/jcm13154554)
Supplement: Supplementary file 1 [file jcm-13-04554-s001.zip › jcm-3110448-supplementary.pdf]

SUPPLEMENTARY MATERIAL

Supplementary Tables:

**Table S1. Causes of Reintervention**		
**Variable**	**Men (n= 34)**	**Women (n=24)**
Endocarditis	6 (17.6)	4 (16.7)
Prosthetic thrombosis	0	2 (8.3)
Pseudoaneurysm	6 (17.6)	2 (8.3)
Abdominal aortic aneurysm	4 (11.8)	2 (8.3)
Prosthetic degeneration	4 (11.8)	2 (8.3)
Failed Aortic Repair	4 (11.8)	3 (12.6)
Myxoma	0	2 (8.3)
Dissection	3 (8.9)	2 (8.3)
New endovascular intervention	6 (17.6)	4 (16.7)
New ascending aortic aneurysm	1 (2.9)	1 (4.2)
Variables are represented as mean ± standard deviation for quantitative variables and number (%) for categorical variables**.**		

**Table S2. Causes of Death**		
**Variable**	**Male (n=30)**	**Female (n=14)**
**Perioperative period**		
Cardiogenic Shock	12 (40)	6 (42.9)
Hemorrhagic Shock	2 (6.7)	4 (28.5)
Infection/sepsis	10 (33.3)	2 (14.3)
Others	6 (20)	2 (14.3)
**Follow-up**		
**Variable**	**Male (n=66)**	**Female (n=20)**
Heart Failure	15 (22.7)	3 (15)
Stroke	6 (9.1)	0
Infection/Sepsis	8 (12.1)	2 (10)
Cancer	20 (30.3)	4 (20)
Acute aortic syndrome	2 (3)	1 (5)
Sudden Death	2 (3)	1 (5)
Other non-CV causes	13 (19.8)	9 (45)
Variables are represented as mean ± standard deviation for quantitative variables and number (%) for categorical variables**. Abbreviations. CV:** Cardiovascular		

**Table S3. Complications during the hospitalization and follow-up**			
**Variable**	Male (n=506)	Female (n=232)	p
**Hospitalization and 30-day outcomes**			
Permanent Pacemaker	32 (6.3)	14 (6)	0.880
New Onset Atrial Fibrillation	100 (20.2)	44 (19.1	0.746
Reintervention any cause	46 (9.1)	18 (7.8)	0.569
Deaths	30 (5.9)	14 (6)	0.955
**Follow-up**			
Mean Follow-up (months)	53.6 ± 38.7	50.9 ± 36.7	0.368
NYHA ≤ II	402 (98.5)	196 (98)	0.630
LVEF	61.2 ± 53.1	65.5 ± 72.2	0.423
Readmission	22 (4.6)	8 (3.5)	0.508
Reintervention	40 (8.4)	16 (7.1)	0.555
Deaths	98 (19.4)	32 (13.8)	0.065
Variables are represented as mean ± standard deviation for quantitative variables and number (%) for categorical variables**. Abbreviations. LVEF:** left ventricle ejection fraction, **NYHA:** New York Heart Association			

Supplementary Figures

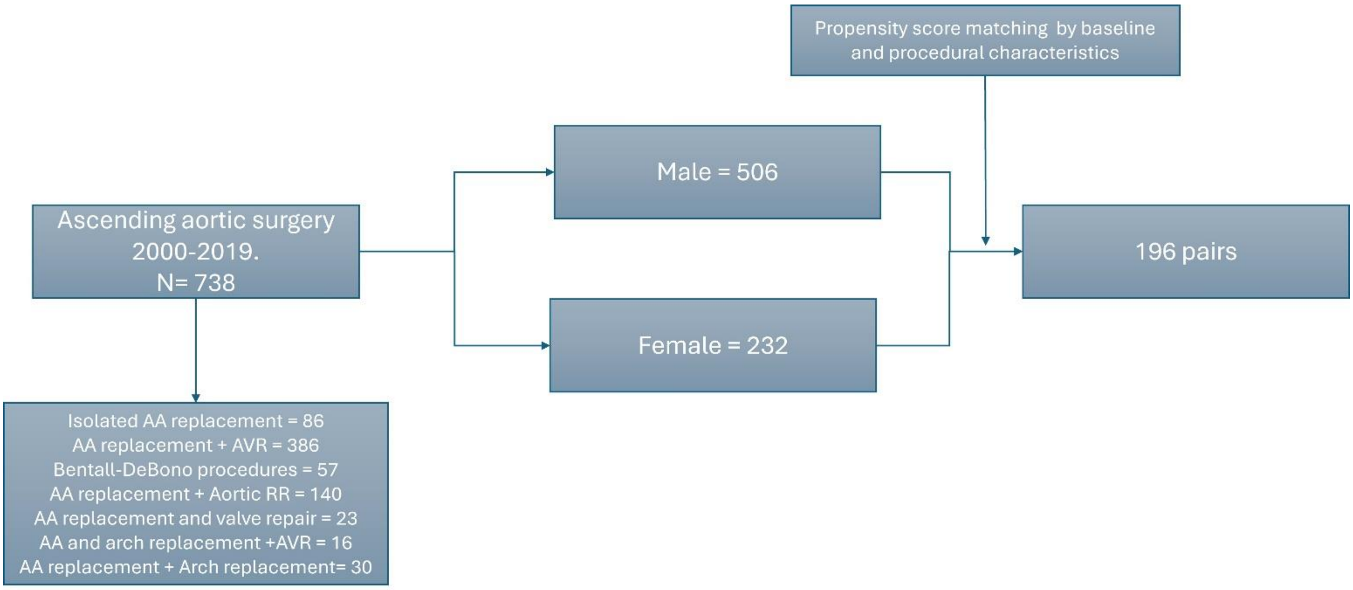

Supplementary Figure S1. Flowchart of the study. Abbreviations: AA: Ascending aorta; AVR: Aortic Valve Replacement; RR: Root Remodeling

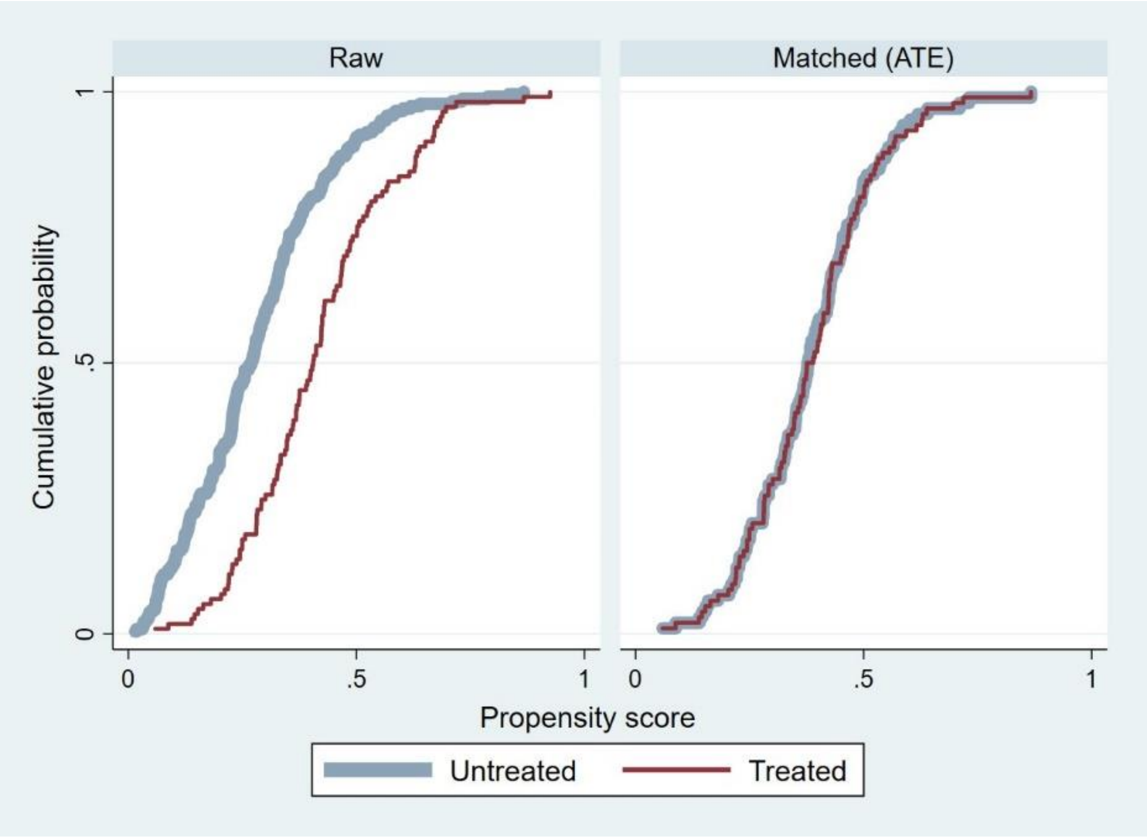

Supplementary Figure S2. Average treatment effect before and after matching showing similar probabilities among men and women.

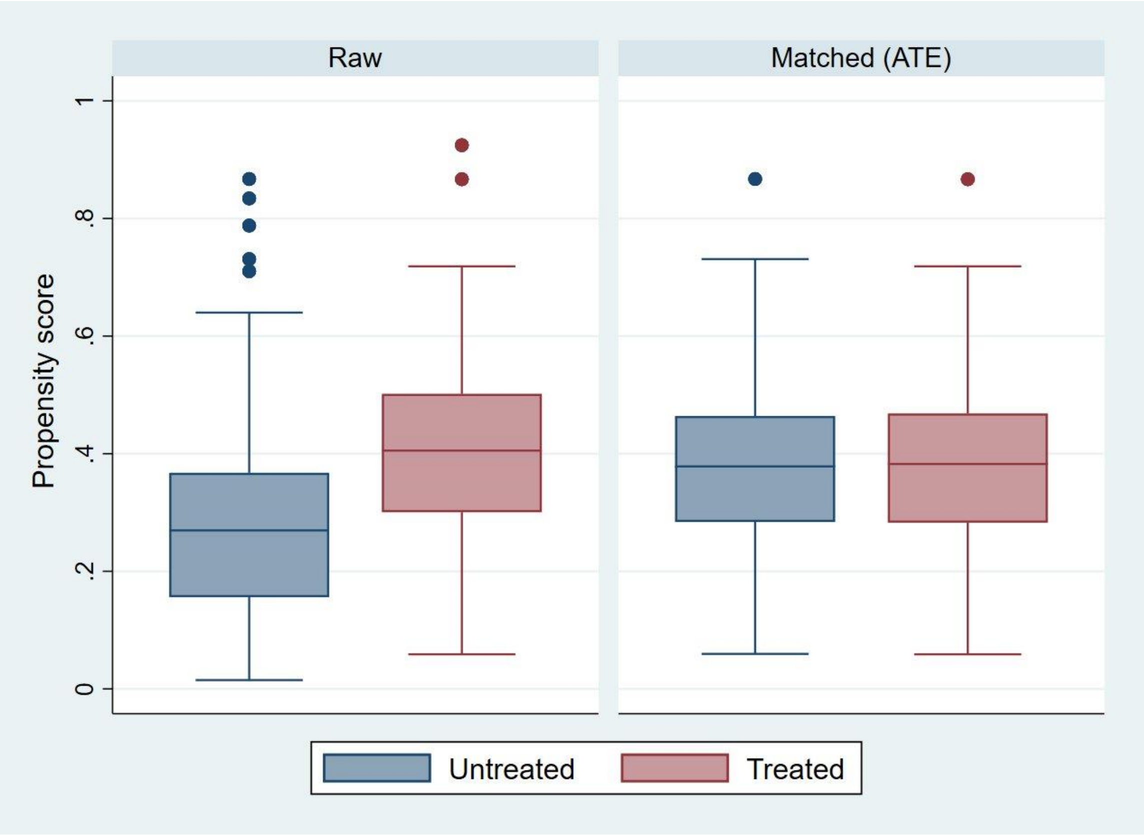

Supplementary figure S3. Box plot of the average treatment effect showing that the propensity score correctly balanced the baseline characteristics after matching
